# The Impact of the Fermentation Strategy on the Flavour Formation of *Ilzer Rose* (*Malus domestica* Borkh.) Apple Wine

**DOI:** 10.3390/foods10102348

**Published:** 2021-10-01

**Authors:** Valerie Ruppert, Georg Innerhofer, Jörg Voit, Peter Hiden, Barbara Siegmund

**Affiliations:** 1 Institute of Analytical Chemistry and Food Chemistry, Graz University of Technology, 8010 Graz, Austria; valerie.ruppert@outlook.at; 2 School for Fruit Growing & Viticulture, Silberberg, 8430 Leibnitz, Austria; georg.innerhofer@stmk.gv.at; 3 Research Centre for Fruit Growing & Viticulture, Haidegg, 8010 Graz, Austria; joerg.voit@stmk.gv.at (J.V.); peter.hiden@stmk.gv.at (P.H.)

**Keywords:** apple wine, heritage apple varieties, maceration, mash fermentation, β-glucosidase, volatiles, sensory evaluation

## Abstract

The flavour and the volatilome of apple wines made from the Austrian heritage variety *Ilzer Rose* was in the scope of this study. The apple wines were produced by adopting oenological practises that are not commonly used in fruit wine production. Different fermentation strategies including the addition of enzymes with β-glucosidase activity, addition of a fining agent, maceration of the mash along with mash fermentation were applied. The volatile compounds of the juices as intermediates and the resulting apple wines were analysed using headspace-SPME GC-MS. CATA technique with a well-trained panel was applied for sensory evaluation. The results show that the flavour of single-variety apple wine can be significantly altered by taking oenological measures. High correlations were found between the results of the analytical investigation and the sensory evaluation. Maceration of the mash leads to an increase in the fruity character of the products, also reflected by significantly higher fruit ester quantities in the wine. During mash fermentation, spontaneous malolactic fermentation was induced leading to a product with new, but thoroughly interesting sensory properties of the apple wine. The results of this study demonstrate that the integration of oenological measures may open a wide field to the development of a high diversity in apple wine flavour.

## 1. Introduction

Apple wine and cider are traditional alcoholic beverages with an alcohol content lower than 8.25%. The European Cider and Fruit Wine Association (AICV) states that within the European Union, cider and fruits wines have some of the fastest growth rates of all alcoholic beverages [1]. This increase in popularity is also reflected by the number of scientific papers that were published on this topic in the past decade. Several recent papers deal with flavour properties and volatile compounds of commercially available products [2,3,4,5], of ciders produced from different apple varieties [6,7], or different stages of maturity [6,8], or cider and apple wines produced with varying production strategies [7,9,10,11,12,13]. In contrast to wine technology, however, in which the impact of grape variety, oenological yeast strains, fermentation strategy or other parameters have been well investigated, there is still a lack of knowledge with respect to the behaviour and the properties of apple wines and ciders. 

In contrast to other European countries, especially non-sparkling apple wine without added flavour has a long tradition in Austria. In many rural areas where the cultivation of pip fruits is an important economic factor, apple wine is a common and wide-spread beverage. In contrast to the production of wine, the complexity of the production of apple wine has been long underestimated, which frequently resulted in highly oxidized and in many cases off-flavoured products. With the recent introduction of oenological techniques in apple process lines (e.g., temperature control during the fermentation, addition of nutrients or sulphites), the character of fruit wines has changed completely, and they have emerged as highly attractive products possessing wine-like character and often showing the typical varietal flavour properties of the processed fruits.

In the southern regions of Austria, particularly in the Province of Styria, apple breeding has a long tradition. Approximately 75% of the Austrian apples are cultivated and harvested in this region. Besides the cultivation of modern and international apple varieties such as *Gala*, *Golden Delicious*, *Braeburn* or *Idared*, a large number of heritage varieties are grown there [14]. Approximately 25% of the heritage varieties are cultivated in meadow orchards in extensive farming regimes on old and mostly large trees that are difficult to harvest. Recently, some interesting heritage varieties were planted in modern plantations making the fruit available in larger quantities for further processing. The specific and in many cases highly attractive flavour properties of these heritage varieties make them an interesting raw material for the production of apple wine and cider with distinct varietal flavour properties. The fact that the major portion of apple wine is not processed in large manufacturing lines but in rural, and thus, relatively small production sites, leads to a large variety of products and also gives high flexibility to the producers. It is also noteworthy that in Austria, apple wines are usually not pasteurized but—In accordance to oenological practices—bottled after the end of the fermentation process.

There is controversy in the literature about the impact of the apple variety and the fermentation strategy on the final flavour of the apple wines [11,15]. In our experience from various studies with modern and heritage varieties, both the variety and also the fermentation process significantly impact the flavour of the final product. It is well known from oenology, that different product characteristics can be evoked with the selection of the yeast strain and the fermentation parameters. A recent Austrian study demonstrated that the production of single-variety apple wines fermented under standardized conditions leads to products that significantly differ from one another in their characteristics [16]. Like sommeliers or wine experts, apple wine experts are also able to recognize the apple variety from the corresponding apple wine.

One of these heritage varieties is the *Ilzer Rose* apple, which is a variety that was described for the first time more than 120 years ago with its origin in the village Ilz. *Ilzer Rose* apples are rather small, red-cheeked, white-fleshed apples with a pronounced rose-like flavour which makes them particularly attractive for the production of apple wine [14]. The *Ilzer Rose* volatilome was recently investigated. Interestingly, in addition to the well-known apple volatiles, many terpenoids were identified, especially in the skin of this apple variety [17]. In several grape varieties such as Muscat grapes, approximately 90% of the terpenes are glycosidically bound [18]. Thus, it is common practice to add enzymes with β-glucosidase activity to release higher amounts of terpenes upon enzymatic cleavage from the glycone and, as a consequence, to impact the flavour of the resulting wine due to an increase in the amount of free terpenes. In a recent study of our group, the treatment of the *Ilze Rose* mash with an oenological enzyme possessing pronounced β-glucosidase activity led to a concentration increase in terpenoids such as linalool, however, the overall linalool concentration was too low to impact the flavour of *Ilzer Rose* apple wine [19]. 

In this study on the flavour of *Ilzer Rose* apple wine, some common oenological practices (i.e., application of different enzymes that are commonly used in wine technology, maceration, mash fermentation) were transferred from oenology to the production of *Ilzer Rose* apple wine in order to gain (i) deeper understanding of the flavour and volatile compounds of the products and to (ii) investigate the impact of technological/oenological practices on the development of *Ilzer Rose* apple wine flavour. On the global wine market, a new type of wine was introduced approximately a decade ago [20,21]. Wines of this type are known as ‘orange wines’ or ‘natural wines’ and they are produced in a manner similar to that used in the ancient Georgian traditional Qvevri wine-making method [22]. Even though the classification of the term ‘orange wine’ does not seem to be completely clear [23], ‘orange wines’ have recently gained popularity among wine consumers. In contrast to modern oenological practice, in which white grapes are pressed immediately after the harvest followed by immediate fermentation of the grape juice, to produce ‘orange wines’ the white grapes are macerated and fermented in direct contact with the mash which results in wines with completely different properties [21,24]. In this study, we transferred this concept to *Ilzer Rose* apple wine production and investigated the final product after apple mash fermentation. With the knowledge that volatiles such as terpenoids are enriched in the apple skin, apple wines with interesting properties were expected.

In order to gain a deep understanding of these *Ilzer Rose* apple wines on a molecular and a sensory basis, we applied analytical techniques that are common in flavour chemistry (i.e., headspace solid phase micro extraction coupled to gas chromatography mass spectrometry, HS-SPME GC-MS) together with techniques from modern sensory science (i.e., check all that apply, CATA). We hypothesize that the application of the described oenological measures will significantly impact the flavour of *Ilzer Rose* apple wine. The results will not only help to deepen the knowledge of the product, but also to assist fashioning apple wine flavour and to make these products even more attractive to consumers.

## 2. Materials and Methods

### 2.1. Fruit Material

The apples of the heritage apple variety *Ilzer Rose* were cultivated in the orchards of the School of Fruit Growing & Viticulture, Silberberg in the south of Austria following the European guidelines for integrated pest management IPM. A total of 157 kg *Ilzer Rose* apples was harvested on 1 October 2018. After the harvest, the apples were transported to the Research Centre for Fruit Growing & Viticulture, Graz-Haidegg where the fruits were stored for two weeks at approx. 15 °C to promote postharvest ripening and flavour development.

### 2.2. Preparation of Juice Samples

**Figure 1:**
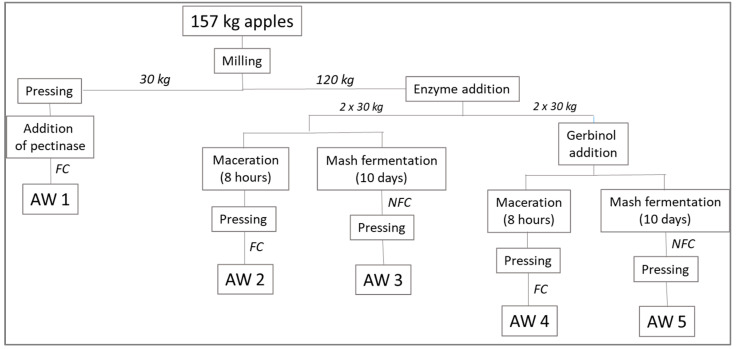
Production of *Ilzer Rose* apple wines using different fermentation strategies—Overview, FC fermentation control, NFC no fermentation control.

Juice preparation and the subsequent apple wine (AW) production was carried out at the Research Centre for Fruit Growing & Viticulture, Graz Haidegg in special micro fermentation devices. Figure 1 gives an overview of the processing steps for the five apple wines. The apples were ground using a pome fruit grinder (Speidel, Ofterdingen, Germany) and the crushed apples were divided into portions of 30 kg each. For the pressing, a hydraulic press with an initial pressure of 2 bar was used. To achieve maximum yield, repressing was carried out twice, at 2.5 and 3 bar.

To follow the formation of volatiles in dependence of the maceration time prior to the fermentation process, the following juice samples were prepared and analysed: (i) juice taken immediately after the pressing procedure without maceration, (ii) juice samples taken after 2, 5 and 8 h maceration of the mash before the fermentation process was started. For the analysis of the volatiles, the samples were immediately deep frozen and stored at −26 °C until further use.

### 2.3. Preparation of the Apple Wines

Apple wine 1 (AW1) was produced as a reference product without maceration; the mash was pressed immediately after milling of the apples. Pectinase (Enzym MS flüssig, Prezis; 5‰) was added to the juice for AW1 for clarification. Ascorbic acid (100 mg L^−1^) was added to the juice prior to fermentation as antioxidant. For the production of apple wines AW2 to AW5, a pectolytic enzyme with β-glucosidase activity (Trenolin^®^ Bouquet PLUS, Erbslöh, Geisenheim, Germany) was added to the mashes; additionally, sulphites (Solution Sulfureuse; Erbslöh, Geisenheim, Germany; 30 mg L^−1^) were added to protect a clean apple wine aroma. For the production of AW2 and AW4, the mash was allowed to macerate for 8 h prior to pressing and fermentation. For the production of AW4 and AW5, the fining agent Gerbinol^®^ CF (Erbslöh, Geisenheim, Germany; 2‰) was added to the mash with the purpose of binding unbalanced tannins and for clarification. For the apple wines AW3 and AW5, mash fermentation (10 days) was performed in accordance with ‘orange wine’ production strategies. The residual mash was pressed after the fermentation.

*Saccharomyces cerevisiae* var. *bayanus* (LALVIN^®^ EC-1118; Lallemand Inc., Montreal, QC, Canada; 250 mg L^−1^) was added to all approaches to initiate the fermentation. Fermentation was controlled for AW1, AW2 and AW4 (‘FC’, temperature control (15.5 °C); addition of two doses of thiamine and diammonium phosphate (Vitamon Liquid, Erbslöh, Geisenheim, Germany; 0.3 ‰) as nutrients during the fermentation; addition of bentonite 2.5 g L^−1^); for AW3 and AW5 temperature was not controlled nor were nutrients added (‘NFC’). At the end of the fermentation, samples for GC-MS analyses were drawn and deep frozen at −26 °C until the GC analysis. Sulphites were added as antioxidant (Sulfureuse Solution, Erbslöh, Geisenheim, Germany; 75 mg L^−1^) to the apple wines after the fermentation to stabilise the samples until sensory evaluation. After sedimentation of turbid particles, the apple wines were filtered (sheet filter, 150 K, Seitz, Rechberghausen, Germany) and bottled into 500 mL glass bottles with screw caps. The bottled apple wines were stored in a cooling room in the dark until further use.

### 2.4. Analysis of Basic Fruit Wine Parameters

#### 2.4.1. Sugar Concentration

The sugar concentration was tracked starting with apple juice and was monitored throughout the entire fermentation process. A portable digital density meter (DMA35, Anton Paar, Graz, Austria) was used and the sugar concentration was determined as °Brix. The residual sugar concentration of the final products was performed via FTIR analysis following the OIV/OENO Resolution 390/2010 (FOSS-WineScan, Foss, Hamburg, Germany) [25].

#### 2.4.2. Acidity

The total acidity was analysed as equivalents to tartaric acid by titration of a defined volume of juice or apple wine, with blue caustic solution (0.1 M NaOH with bromothymol blue BTB as colour indicator) to reach a colour change from green to blue at pH 7. In addition, the pH values of the final products were measured using a standard pH electrode. The determination of malic acid and lactic acid in the final products was performed via FTIR analysis following the OIV/OENO Resolution 390/2010 (FOSS-WineScan, Foss, Hamburg, Germany) [25].

#### 2.4.3. Sample Preparation and GC-MS Analysis

Headspace solid phase microextraction (HS-SPME) followed by gas chromatography mass spectrometry (GC-MS) was used for the investigation of the juices and apple wines after optimising the analytical procedure (details on method development not shown). For the analysis of volatiles from the apple juice, 10 mL of juice were mixed with 4 g of NaCl. An aliquot of 200 µL was transferred into a 20 mL headspace glass vial. For the apple wines, 50 µL of apple wine was transferred into a 20 mL headspace vial with the addition of 50 mg NaCl. In all cases, 2-octanol was added as an internal standard (10 ng absolute; Sigma-Aldrich, Vienna, Austria; purity ≥ 98%). The extraction/enrichment of the volatiles was performed by headspace solid-phase micro extraction (HS-SPME) using a CTC Combi PAL sampler (CTC Analytics, Zwingen, Switzerland). A 50/30 µm DVB/Car/PDMS 2 cm stable flex SPME fibre (Supelco, Bellefonte, PA, USA) was used for the enrichment of the volatiles. Prior to the extraction of the volatiles, the samples were equilibrated in the oven of the autosampler at 40 °C for 5 min, followed by a 20-min exposure of the SPME fibre to the headspace of the samples. Samples were stirred thoroughly during the equilibration and enrichment process. Immediately after the exposure, the fibre was transferred into the injector of the gas chromatographic system for thermo-desorption. The SPME fibre was left in the injection port for re-conditioning (20 min) before it was exposed to the headspace of the next sample.

The GC-MS analysis was conducted on two GC columns of different polarities of the stationary phases. For both column types, GC-MS chromatograms were recorded on Shimadzu GC-2010 Plus, MS QP 2020 (Shimadzu Europa GmbH, Duisburg, Germany). For the semi-polar column (Rxi^®^-5 ms, 30 m × 0.25 mm × 1 µm, Restek Corporation, Bellefonte, PA, USA) the following conditions were used: temperature program starting at −10 °C for 1 min with a temperature ramp of 8 °C min^−1^ up to 280 °C (holding time 12 min). The injector temperature was 270 °C, splitless injection was applied. Special SPME liners (constant inner diameter of 0.75 mm) were used in the GC injection systems. Cryo-focussing by blowing liquid nitrogen into the GC-oven was applied to reach the start temperature of −10 °C with the aim of obtaining higher resolution and better peak shape for compounds with very high volatility. Helium was used with a linear velocity of 31 cm s^−1^ (constant flow). The mass selective detection was performed in the scan mode (35–350 amu, EI (70 eV), interface temperature 270 °C, ion source temperature 230 °C). For the polar column (ZB-Wax Plus, 20 m × 0.18 mm × 0.18 µm; Restek Corporation, Bellefonte, PA, USA) the following conditions were used: temperature program starting at 40 °C for 1 min with a temperature ramp of 8 °C min^−1^ up to 240 °C (holding time 3 min). The injector temperature was 250 °C, splitless injection was applied. Helium was used with a linear velocity of 35 cm s^−1^ (constant flow). The mass selective detection was performed in the scan mode (46–250 amu, EI (70 eV), interface temperature 200 °C, ion source temperature 220 °C).

All samples were analysed threefold or fourfold in randomized order. Data deconvolution and integration of the deconvoluted peaks was performed with PARADISe software, based on the PARAFAC2 model [26,27]. After the deconvolution procedure, the identification of the volatiles was based on probability-based matching of their mass spectra with those from MS libraries (NIST14, Adams Essential Oils library, FFNSC 3), authentic reference compounds and linear temperature-programmed retention indices. Linear-temperature programmed retention indices (RI) were calculated by analysing the homologous series of n-alkanes (C_6_–C_26_) using the same GC-MS conditions as for the samples. Compounds were considered as identified upon correspondence of the mass spectra and when their RIs on the semi-polar compound fit to the RIs from retention index databases (in-house RI database based on authentic reference compounds, http://www.flavornet.org or http://webbook.nist.gov/chemistry; last access 27 September 2021). RI on the polar compounds were used as criterion to confirm the identity of the compounds (detailed data are not listed in this paper). Semi-quantification of the investigated compounds was performed by comparison of the peak areas obtained from total ion chromatograms after the deconvolution process against those of the internal standard 2-octanol using a response factor of one for each compound.

#### 2.4.4. Sensory Evaluation

For sensory evaluation, a trained panel was used. The panel consisted of 15 panellists (11 females, four males). All subjects had been trained and selected according to DIN EN ISO 8586 [28]. In addition, the panellists had long-standing sensory experience on different food matrices. Sensory evaluation was performed in a sensory laboratory under standardised conditions. To sensitize the panellists for the odours that were expected from this study, the subjects were specifically trained on odour active compounds with fruity, green and floral notes. The selection of the compounds was also based on the results from the analysis of the volatilome. All compounds (linalool, phenylethyl alcohol, rose oxide, geranyl alcohol, α-farnesene, (*E*) 2-hexenal, hexyl butanoate, 2-hexen-1-ol, phenyl acetate, hexanol, (*Z*) 3-hexenol, β-damascenone, linalool oxide, methyl dihydrojasmonate, acetoin, (*Z*) 3-hexenal, 2-methyl hexylbutanoate, nonanal, hexyl acetate, 2-ethylhexanol, benzyl alcohol, α-terpinene, p-cymene, benzaldehyde, bisabolene, phenethyl acetate, β-ionone, geraniol, citronellol, citral; Sigma-Aldrich, Vienna, Austria; purity > 95%, food grade quality) are registered in the European Union as flavouring compounds and were authorized to be used as food flavourings according to regulation (EU) No 872/2012 at the time of the investigations. Filter strips were dipped into 1% *v*/*v* ethanolic solutions of a single compound and put into transparent covers after ethanol evaporation until sensory evaluation (descriptive analysis) started (13–15 odours presented in one session).

A list of 28 descriptors was created for the evaluation of the apple wines. The selection of the descriptors was based on descriptive analysis and subsequent discussion of the descriptors for the samples prior to CATA analysis (data not shown). The main attributes were selected and potentially grouped together. The following attributes were used: fresh/fruity, complex fruits, exotic fruits, apple flavour, apple skin, citrus, floral, rose, honey, green/grassy, herbs/spicy, woody, musty, earthy, phenolic, oxidative notes, wine-like, yeast notes, fermentative notes, solvent/pungent, low sweetness, high sweetness, inexpressive/bland, low acidity, high acidity, bitter, tannic, full-bodied.

The evaluation of the apple wines was designed and carried out using Compusense software (Compusense Inc.; Guelph, ON, Canada). Blind tasting was performed; all samples were coded with random three-digit numbers. Randomisation of the samples was accomplished by using the Latin square design. The apple wine samples (sample amount 70 mL; room temperature) were presented in standard wine tasting glasses with lids. Panellists were asked to open the lids only immediately before the evaluation of the respective sample. The subjects had to evaluate all samples in one session in accordance with the given sample order. For the purpose of quality assurance, one sample was offered in duplicate to evaluate the reproducibility of each single panellist. The attributes were shown on the screen in randomised order and the subjects had to tick on all qualities that applied to the respective sample. Between the evaluation of different samples, the assessors were asked to neutralize the oral cavity with tap water. Results given by single panellists were summarised in a contingency table. These data were statistically processed using correspondence analysis.

#### 2.4.5. Statistical Analysis

To observe statistically significant differences between the relative concentrations of volatiles in the apple wines that were produced with different fermentation strategies, 1-one-way analysis of variance (ANOVA, *p* = 0.05) was applied. In case, statistically significant differences were observed within the data sets, the ANOVA was followed by post-hoc pairwise comparison using Tukey’s honestly significant difference (HSD) test control (*p* < 0.05) to check for differences between single samples. To identify possible correlations between samples and volatiles, principal component analysis (PCA) was conducted using Pearson correlation. For the evaluation of results obtained from CATA experiments, correspondence analysis with Cochran’s Q test (*p* = 0.05) was applied with subsequent multiple pairwise comparison applying the McNemar test with Bonferroni correction. Principal coordinates analysis (PCoA) was applied to determine the correlation coefficients for visualisation in a two dimensional map. For all statistical analyses the MS Excel add-in XLSTAT was used (Addinsoft (2019), XLSTAT statistical and data analysis solution. Long Island, NY, USA).

## 3. Results and Discussion

In this study, we investigated the volatile compounds and sensory properties of apple wine produced from the Austrian heritage apple variety *Ilzer Rose* after applying different fermentation strategies. As in all food commodities, high quality starting material is essential to produce a high-quality end product. In the specific case of apples, an advanced ripening stage of the fruit is required. Postharvest ripening of apples generally leads to the formation of significantly higher concentrations of esters which are the flavour determining primary flavour compounds. The formation of these takes place upon binding of the ripening phytohormone ethylene to the ethylene binding receptors of the fruits mainly during postharvest ripening. A recent study demonstrated that the apple variety and degree of ripeness significantly impact the flavour of the final product [11]. Based on this knowledge and the personal experience of local apple breeders, the *Ilzer Rose* apples were allowed to ripen for approximately two weeks after the harvest which led to a significantly more pronounced flavour of the fruits (results not shown).

The analysis of the volatile compounds of the apple juices (Section 3.1) and apple wines (Section 3.2.2) was performed by applying HS-SPME GC-MS. The suitability of this straight-forward technique for this purpose has been demonstrated previously. It was not the aim to fully quantify the volatiles in the juices and the fruit wines, but to investigate the impact of the oenological measures on the flavour formation. As a consequence, semi-quantification as described by Elmore, 2015 was performed [29].

### 3.1. Volatile Compounds Obtained from Apple Juice and Dependence on the Maceration Time

The first step in the traditional production line for apple wines is the production of apple juice prior to fermentation. In traditional Austrian rural fruit processing companies, it is common practise to start the fermentation procedure immediately after pressing, with the exception of the potential addition of pectolytic enzymes to the mash to increase the juice yield, in particular for fruits that have been stored for a longer period of time. In contrast, it is common practice in wine technology to perform maceration of the grapes with or without addition of specific enzymes to extract valuable components from the fruit flesh or the skin into the must before fermentation [18,30]. In many cases, enzymes with glycosidic activity are added to release glycosidically bound aroma compounds into the grape juice. In this study, we followed this practice by adding an oenological enzyme with pronounced β-glucosidase activity to the apple mash and following the development of volatile and potentially aroma active compounds in the juice over a period of eight hours. We were unable to confirm our initial assumption that this would lead to an increase in the floral, rose-like flavour in *Ilzer Rose* juice and wine, in a manner similar to the release of terpenoids from their glycosides as observed in aromatic grape varieties. The observed increase in terpene-concentrations upon enzymatic treatment was far too low for a significant impact of the overall aroma [19]. Nevertheless, the maceration of the apple mash with the addition of β-glucosidase led to a significant change in the volatilome of the *Ilzer Rose* apple juice. A total of 70 volatile compounds was identified in the juice (Table 1). The volatilome is dominated by alcohols, esters, short chain fatty acids and carbonyl compounds. The relative concentrations of the compounds given in Table 1 clearly show that maceration of the mash prior to pressing already impacts the composition significantly after two hours of maceration with a further increase in concentration for several compounds during longer maceration times. Strong increases could be observed for esters (8.5-fold increase in the total ester concentration within 8 h of maceration) and alcohols (threefold increase in total alcohol concentration after 8 h of maceration), whereas for carbonyls, free acids and terpenoids only small concentration changes were observed. The highest concentration increases were found for the C_6_ alcohols 1-hexanol, 3-hexenol (*Z*) and 2-hexenol (*E*), for the corresponding acetates and the hexyl esters hexyl hexanoate and hexyl 2-methylbutanoate. The observed increase in concentration is not, however, derived from their release from glycosides, but most probably from the lipoxygenase pathway upon cell disruption and contact with oxygen and the acidic catalysed formation of the corresponding esters in the mash. Having the odour properties of these compounds in mind (i.e., fresh green, grassy, leafy for the alcohols and green, fresh, fruity, apple- and pear-like for the mentioned esters), their concentration increase will impact the fruity character of the juice that is further processed. Consequently, the mash was macerated for eight hours to produce apple wines AW2 and AW4, to take advantage of these reactions.

### 3.2. Investigation of the Apple Wines

#### 3.2.1. Fermentation and Basic Apple Wine Parameters

The fermentation of the *Ilzer Rose* juice was carried out in specifically configured micro fermentation devices suitable for controlled pilot-scale fermentation of wine and fruit wines. To be able to evaluate the impact of the investigated fermentation strategies only, some measures were taken to achieve standardized starting conditions (i.e., addition of *Saccharomyces cerevisiae* var. *bayanus* as starter culture for the alcoholic fermentation, sulphite addition to the juice/mash to protect the clear apple aroma). AW1 that was regarded as reference product was fermented according to modern practices in apple wine production. Specific oenological measures were taken for the apple wines AW2 to AW5 as described in Section 2.3. The fining agent Gerbinol^®^ was added to AW4 and AW5 to potentially bind polyphenol-degradation products. Fermentation was controlled with respect to temperature and nutrients for AW1, AW2 and AW4, whereas for AW3 and AW5 the oenological practices that are common for ‘orange wine’ production were followed; hence, temperature was not controlled, nor were nutrients added.

**Figure 2:**
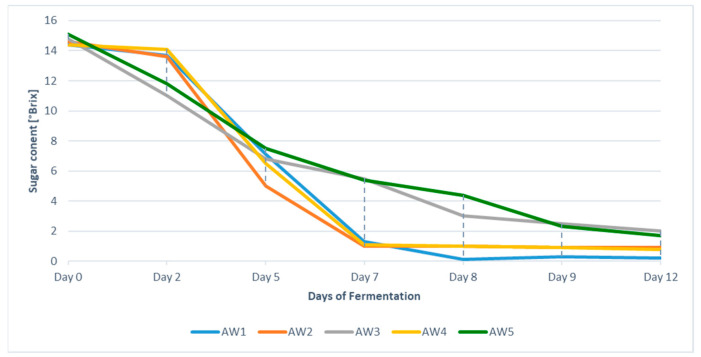
Concentrations of the total amount of sugars [°Brix] in apple wines AW1 to AW5 followed along the fermentation process.

The fermentation processes were monitored over a period of 12 days. Figure 2 shows the sugar concentrations/total solubles in terms of °Brix in all products. Without the addition of sulphites in AW3 and AW5, the metabolism of the yeast started quicker than in the other three approaches. After day 3, the reaction rates increased for AW1, AW2 and AW4 in comparison to the mash fermentation of AW3 and AW5. The final total quantity of solubles remained highest for the mash fermented products and lowest for the reference apple wine (Figure 2). As we aimed to evaluate the impact of the fermentation strategy without the impact of any reactions occurring during maturation of the wine, turbidity was allowed to sediment after the end of the fermentation followed by sheet filtration to eliminate the fine lees. Subsequent bottling in glass bottles with screw caps and cold storage of the apple wines until the sensory analysis was applied to avoid oxygen impact and to decelerate ageing reactions as far as possible. 

The characteristics of the final products are given in Table 2. The average alcohol content was 7.3% with no significant differences between the five apple wines. All other investigated parameters showed differences depending on the fermentation strategy. Residual sugars were lowest in AW1 with a concentration of <1.7 g L^−1^, whereas for AW2 and AW4 slightly higher residual sugar concentrations were observed. Interestingly, the mash fermented apple wines AW3 and AW5 showed significantly higher concentrations in residual sugars (approx. 8.2 g L^−1^). These differences in concentration were already expected after monitoring the fermentation process (Figure 2). With respect to the total titratable acids, the highest values were observed in the products AW2 and AW4 where maceration of the mash was performed, followed by the product AW1 with straight-forward fermentation. As for the total acidity, the malic acid concentrations were in a comparable range for products AW1, AW2 and AW4. By contrast, the mash fermented apple wines AW3 and AW5 showed significantly lower total acidity and lower malic acid concentrations; in contrast to this, lactic acid formation was observed in the mash fermented apple wines only. These results indicate, on the one hand, a degradation of acids via the Krebs cycle upon the mash fermentation. On the other hand, the presence of lactic acid in AW3 and AW5 shows that spontaneous malolactic fermentation took place as a secondary fermentation leading to deacidification of these fruit wines. Growth of naturally occurring lactic acid bacteria inducing spontaneous malolactic fermentation is frequently observed in oenology. Besides a change in the acid composition of the fruit wine, impact of malolactic fermentation on the formation of volatile compounds as well as on the sensory properties has to be expected [31].

#### 3.2.2. Volatile Compounds from Apple Wines

A total number of 57 volatile compounds (4 acids, 14 alcohols, 2 carbonyls, 34 esters, 3 terpenoids, Table 3) could be identified in the apple wines produced when applying different fermentation strategies. In the literature, substituted phenols are reported as part of apple wine flavour [15,32]. Interestingly, phenolic compounds were not identified as part of the *Ilzer Rose* apple wines.

**Figure 3:**
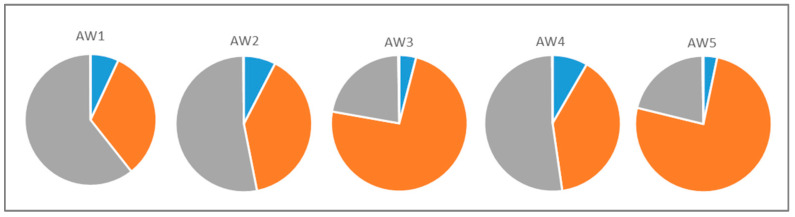
Relative amounts of the compound classes with highest relative concentrations in the apple wines AW1 to AW5; ● ● esters, ● ●acids, ● ● alcohols.

The results of this study clearly demonstrate that the fermentation strategy significantly impacts the final amounts of volatiles in the apple wines and their relation to one another. Strong dependence on the fermentation strategy was observed for esters, alcohols and free acids, whereas carbonyl compounds and terpenoids showed only small concentration differences in the five different fruit wines. Figure 3 gives a comparison of the relative proportions of esters, alcohols and acids in the apple wines AW1 to AW5. From Figure 3 and from data given in Table 3, it can be seen very clearly, that the fermentation strategy significantly impacts the volatilome, whereas the addition of the fining agent Gerbinol^®^ to AW4 and AW5 does not significantly influence the composition of the volatilome.

The volatile compounds in fermented products are, on the one hand, determined by the volatilome of the starting material; on the other hand, the secondary metabolism of the used strain is an important source for the volatile compounds in the final product. Furthermore, the composition of the volatile fraction can be influenced by the selection of the yeast genera and by the nutrients that are available for the microorganisms. Two recent articles on *Saccharomyces cerevisiae* summarize the role of the respective enzymes on the biosynthesis of secondary metabolites in the course of the fermentation process [33,34].

Higher alcohols (fusel alcohols) as reaction products from the corresponding amino acids formed via the Ehrlich pathway belong to the most abundant volatile compounds in fermented beverages. In our study, 2-methyl butanol, 3-methyl butanol, 1-pentanol and phenylethyl alcohol were present in partly very high relative concentrations, with a highest average relative concentration of approx. 3760 µg L^−1^ for 3-methyl butanol and the lowest average relative concentration of 7.7 µg L^−1^ for 1-pentanol. Even though the concentration differences were statistically significant, with the exception of 3-methyl butanol, the effect of the fermentation strategy on compound concentrations was only minimal. The same behaviour was observed for the corresponding acids, 2-methyl- and 3-methyl butanoic acids. By contrast, for the corresponding acetates (2-methyl- and 3-methyl butyl acetate, phenethyl acetate), and also the straight-chain acetates ethyl acetate, propyl acetate and hexyl acetate, significantly lower concentrations were observed in the mash fermented apple wines AW3 and AW5 than in the other fruit wines. Alcohol acetyl transferases are required to catalyse the biosynthesis of acetate esters from the corresponding alcohols. The observed behaviour indicates a down-regulation of alcohol acetyl transferase activities in AW3 and AW5 as no nutrients were provided during mash fermentation. Highest concentrations of the mentioned acetates were found in AW1; the quick start of the fermentation without maceration nor mash fermentation and, thus, sufficient energy supply for the yeast to catalyse the acetate formation might be the reason for this behaviour.

All other esters that were identified in the apple wines belong to the group of fatty acid esters (mainly ethyl or methyl esters of straight chain fatty esters C_3_ to C_12_ or of methylated acids). These esters are of particular importance for the flavour of apple wines as their sensory properties are described as fruity, apple- or pear-like, pineapple or strawberry like, depending on the chain length. The biosynthesis of esters mainly occurs intracellularly by the fermenting yeasts and is followed by a diffusion through the yeast’s plasma membrane [35]. As a consequence, their formation is strictly controlled by the activities of the related yeast alcohol acetyl transferases. Straight-chain fatty acids are released from the cytoplasmic fatty acid synthase complex; the subsequent formation of fatty acid esters is a result of the reaction of the fatty acid with the acyl-CoA component of ethanol [33,34]. As discussed in the previous paragraph, the methylated acids are reaction products of the amino acid degradation via the Ehrlich pathway; the subsequent ester formation requires ethylation with ethanol. According to the literature, little is known about the enzymatic control of the ethylation of alkylated acids. With respect to the regulation of enzymes catalysing the esterification processes, specifically little is known about their behaviour in natural grape or fruit wine [34]. However, an increase in the initial nitrogen content together with lower fermentation temperatures seem to favour ester formation [36]. We observed significantly lower overall ester concentration in the mash fermented apple wines AW3 and AW5 compared to the apple wines AW1, AW2 and AW4. Obviously, the addition of nutrients along with temperature control are the reasons for the formation of significantly higher concentrations of esters in the apple wines AW1, AW2 and AW4.

Isoamyl lactate was determined in remarkable quantities in the mash fermented apple wines AW3 and AW5, but not in the products from other approaches. Isoamyl lactate is a reaction product from malolactic fermentation that was observed in the mash fermented fruit wines. With respect to terpenoid compounds, a significant, but small increase in concentrations was observed between AW1 and the other four apple wines with little concentration differences between AW2 to AW5. As reported previously [19], we assume a release of the terpenoids from their glycosidic form; however, their overall concentrations are too low to impact the flavour of any of the four apple wines.

**Figure 4:**
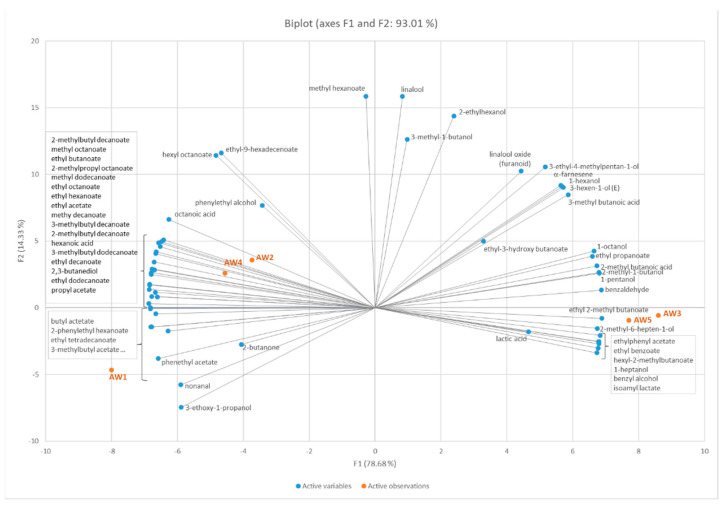
PCA plot (Pearson correlation) showing the correlation of the volatile compounds and the apple wine samples.

Multivariate statistical analysis (PCA analysis) was performed on the volatile compounds to make the correlations between volatiles and fermentation strategy more clearly visible and to demonstrate similarities and differences between the products (Figure 4). High correlations were found between AW2 and AW4 as well as between AW3 and AW5, again demonstrating that the impact of the fining agent Gerbinol^®^ is negligibly small. AW1 is separated in the PCA plot from all other apples wines. However, a higher correlation with AW2 and AW4 is observed than with AW3 and AW5. This indicates certain similarities these products share. A strong correlation was found for AW2 and AW4 with a long list of esters possessing fruity character. This demonstrates that the maceration of the mash for a period of eight hours increased the release of free fatty acids from the mash or favoured fatty acid biosynthesis by the yeast. The mash fermented products do not show correlations with fruit esters, but with some alcohols and reaction products from the Ehrlich pathway and malolactic fermentation.

#### 3.2.3. Sensory Analysis

**Figure 5:**
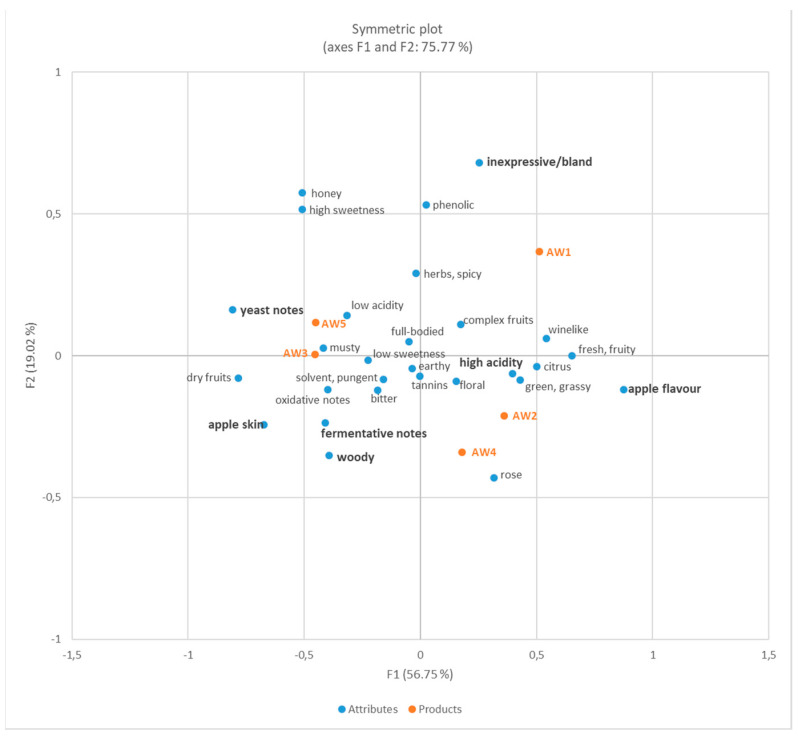
Biplot (PCoA) as a result of the correspondence analysis performed on the data from CATA analysis, significant attributes (threshold 0.1) are printed in bold.

In addition to the analysis of non-volatile and volatile compounds, we aimed to investigate the impact of different fermentation strategies on the sensory properties of the resulting *Ilzer Rose* apple wines. As we were not interested in the appeal of the products to consumers, but in a detailed sensory characterization of the products, we worked with a well-trained panel. Due to the well-known limitations of sensory techniques that are based on intensity scoring, we decided to apply CATA which is a citation frequency-based method. The suitability of CATA for product characterization with the use of trained panellists has been described in the literature previously [37,38]. The selection of appropriate descriptors is regarded as essential. Descriptors were collected from the panellists during the pre-study and training phase. The final list of descriptors contained no attributes referring to liking or preference and consisted solely of strictly analytic descriptors that were considered as important for apple juices and apple wines. Prior to the statistical analysis, one attribute (‘exotic’) was removed from the list as it was not ticked in any individual protocol. Figure 5 shows the results of the sensory evaluation in terms of a biplot after correspondence analysis. As from the multivariate analysis of the volatile compounds (Figure 4), a clear differentiation of the apple wines was obtained by CATA analysis. The attributes with the highest significance and thus, the highest impact on the classification of the apple wines, were the attributes ‘apple flavour’, ‘high acidity’, ‘inexpressive/bland’, ‘yeast notes’, ‘apple skin’, ‘fermentative notes’ and ‘woody’. All other descriptors were not discriminative, but they nevertheless deliver valuable information about the products. Similar product groupings were obtained as from the analysis of the volatile compounds (Figure 4). The product AW1 did not show correlations with any of the other apple wines; AW1 showed high correlations with the descriptors ‘inexpressive/bland’, but also with the positively associated descriptors ‘complex fruits’ and ‘wine-like’. As from the results of the volatilome, high correlations—and thus high similarities in flavour—were observed between AW2 and AW4 as well as between AW3 and AW5. The results from sensory evaluation again demonstrate that the addition of the clarifying agent Gerbinol^®^ does not significantly impact the flavour, but that the fermentation strategy (i.e., maceration of the mash and mash fermentation) is the driver for the formation of distinct flavour properties. The maceration of the mash prior to the controlled fermentation of AW2 and AW4 leads to the promotion of ‘apple flavour’ in the wine supported by ‘green/grassy’, ‘citrus’ and ‘floral’ notes. In addition, AW2 and AW4 are strongly correlated with the term ‘high acidity’ which is in accordance with the higher concentrations of acids in these products (Table 2). The biplot in Figure 4 shows a strong correlation of AW2 and AW4 with a large number of fruit esters—their presence seems to be responsible for the expressed perceived fruitiness of AW2 and AW4. The apple wines AW3 and AW5 are strongly correlated with the descriptors ‘fermentation’ and ‘yeast notes’, ‘woody’ and ‘apple skin’. Furthermore, descriptors such as ‘oxidative note’ or ‘dry fruits’ show high correlations with the mash fermented products. In a recent study, the impact of the amount of grape skin during white wine fermentation on the sensory properties of the resulting products was investigated. A similar behaviour—loss in fruitiness and floral notes, loss in acidity, increase in sensory properties not related to fresh fruitiness—was observed in this study [24]. The observed malolactic fermentation in AW3 and AW5 is the reason for perceived low acidity of these two fruit wines.

## 4. Conclusions

In this study, we investigated the flavour of single-variety apple wines produced from the Austrian heritage apple variety *Ilzer Rose* after introducing oenological measures into the production line. Winemakers and oenologists have been aiming in viticulture to emphasise the characteristics of their wines with respect to the flavour properties of the grapes, the vinification, the terroir or the fermentation strategy over the past few decades. As a consequence, the overall wine quality together with the diversity of products on the global market has increased significantly. By contrast, only minor emphasis has been placed on the specific flavour of apple wines. Even though the consumption of apple wines and ciders has greatly increased in recent years, either conventional production regimes have been applied or the products have been heavily flavoured, thus covering the genuine flavour of the original product.

The cultivation of heritage apple varieties offers a large reservoir for raw materials with interesting and highly attractive flavour properties. With this study, we could demonstrate that the introduction of oenological measures to the production of apple wines is a useful tool to (i) optimise and enhance the flavour of the products, and (ii) to introduce new and interesting characteristics as shown by the production of the mash fermented apple wines. We also showed that the application of a fining agent did not result in alteration of the product characteristics. This demonstrates that an uncritical direct transfer of oenological measures into apple wine production might not be useful. Increasing awareness of the large flavour diversity of apple varieties and optimisation of the applied technologies, as demonstrated by this study, may lead to the production of highly attractive fruit wines that do not require the addition of flavouring substances.

## Figures and Tables

**Table 1:** Volatile compounds in *Ilzer Rose* apple juice produced after different maceration times (0 h, 2 h, 5 h, 8 h); quantities are given as mean relative concentrations [µg L^−1^] (*n* = *3*) collected from the headspace using HS-SPME GC-MS calculated by comparison of the peak areas with that of the internal standard 2-octanol with a response factor of 1; SD standard deviation [g L^−1^]; * significant difference among all approaches (*p* ≤ 0,05%), ** highly significant difference among all approaches (*p* ≤ 0.001%).

Compound	RI [DB5] _exp_^§^	RI [DB5] _lit._^$^	Juice 0 h Maceration Mean ± SD [µg L^−1^]	Juice 2 h Maceration Mean ± SD [µg L^−1^]	Juice 5 h Maceration Mean ± SD [µg L^−1^]	Juice 8 h Maceration Mean ± SD [µg L^−1^]	Significance
***acids***							
acetic acid	612	625	1.53 ± 0.3	0.72 ± 0.6	2.56 ± 0.2	1.37 ± 1.2	No
2-methyl butanoic acid	842	856	25.3 ± 1.3 *^a^*	15.9 ± 1.3 *^b^*	17.7 ± 1.5 *^b^*	20.4 ± 0.2 *^a^*^,*b*^	*
hexanoic acid	967	980	3.0 ± 0.0 *^c^*	7.1 ± 0.1 *^b^*	7.0 ± 0.2 *^b^*	10.3 ± 0.3 *^a^*	**
***alcohols***							
1-propanol *^t^*	<600	548	1.94 ± 0.1	2.00 ± 0.7	2.96 ± 0.0	2.36 ± 0.6	No
2-butanol	601	605	0.91 ± 0.0	0.81 ± 0.3	1.25 ± 0.0	1.17 ± 0.3	No
2-methyl Propanol	628	654	2.92 ± 0.1	0.71 ± 0.8	1.20 ± 0.3	1.03 ± 1.1	No
1-butanol	665	660	40.3 ± 3.1 *^b^*	51.4 ± 2.7 *^a^*^,*b*^	60.9 ± 1.6 *^a^*	61.9 ± 2.7 *^a^*	*
1-penten-3-ol	684	686	0.34 ± 0.0 *^b^*	0.92 ± 0.1 *^a^*	0.95 ± 0.0 *^a^*	1.14 ± 0.1 *^a^*	**
2-methyl-1-butanol	739	743	67.5 ± 3.8 *^a^*	84.1 ± 4.5 *^a^*^,*b*^	78.0 ± 0.6 *^a^*^,*b*^	93.6 ± 3.3 *^b^*	*
1-pentanol	767	766	0.36 ± 1.9	3.35 ± 0.5	3.68 ± 0.1	5.07 ± 0.2	No
2,3-butanediol	780	773	1.38 ± 0.4	3.37 ± 1.7	4.31 ± 1.1	4.02 ± 1.1	No
3-hexen-1-ol (*Z*)-	851	858	0.89 ± 0.5 *^c^*	20.5 ± 0.5 *^b^*	21.0 ± 0.1 *^b^*	34.1 ± 2.5 *^a^*	**
2-hexen-1-ol (*E*)-	866	887	12.6 ± 2.1 *^c^*	356 ± 13.9 *^a^*^,^*^b^*	317 ± 11.3 *^b^*	394 ± 19.6 *^c^*	**
1-hexanol	868	867	106.9 ± 0.2 *^b^*	623 ± 43.4 *^a^*	617 ± 23.1 *^a^*	821 ± 60.6 *^a^*	**
1-heptanol	967	970	8.02 ± 1.0 *^b^*	24.0 ± 4.7 *^a^*	27.3 ± 0.4 *^a^*	29.6 ± 0.7 *^a^*	*
1-octen-3-ol	979	979	0.55 ± 0.0 *^b^*	1.93 ± 0.1 *^a^*	1.72 ± 0.0 *^a^*	2.07 ± 0.1 *^a^*	**
methionol	982	980	5.46 ± 0.2 *^a^*	3.91 ± 0.2 *^b^*	3.97 ± 0.3 *^a^*^,^*^b^*	5.12 ± 0.3 *^a^*^,^*^b^*	*
6-methyl-5-hepten-2-ol	993	994	15.6 ± 1.0 *^c^*	29.2 ± 1.5 *^b^*	33.9 ± 0.9 *^a^*^,^*^b^*	40.0 ± 2.2 *^a^*	**
2-ethyl hexanol	1029	1028	2.01 ± 0.1 *^c^*	3.58 ± 0.1 *^a^*	3.00 ± 0.0 *^b^*	3.74 ± 0.0 *^a^*	**
benzyl alcohol	1042	1032	3.04 ± 0.3 *^d^*	8.06 ± 0.2 *^c^*	10.4 ± 0.3 *^b^*	21.5 ± 0.2 *^a^*	**
1-octanol	1069	1069	1.14 ± 0.1 *^d^*	5.14 ± 0.1 *^c^*	7.70 ± 0.1 *^b^*	13.06 ± 0.3 *^a^*	**
3-octen-1-ol (*Z*)-	1071	1047	1.85 ± 0.2 *^b^*	5.96 ± 0.1 *^a^*	6.30 ± 0.2 *^a^*	8.03 ± 0.9 *^a^*	*
phenylethyl alcohol	1127	1113	2.29 ± 0.3 *^c^*	3.12 ± 0.1 *^b^*^,^*^c^*	3.69 ± 0.2 *^b^*	6.04 ± 0.1 *^a^*	**
1,3-octanediol	1264	1275	451 ± 24.3 *^b^*	544 ± 20.3 *^a^*^,^*^b^*	688 ± 24.4 *^a^*	703 ± 71.1 *^a^*	*
1-dodecanol	1477	1475	6.60 ± 2.8	7.69 ± 1.6	6.18 ± 1.4	5.82 ± 1.0	No
***carbonyls***							
2,3-butanedione	615	623	0.15 ± 0.0 *^c^*	0.25 ± 0.0 *^b^*	0.37 ± 0.0 *^a^*	0.39 ± 0.0 *^a^*	**
butanal	602	601	51.2 ± 0.0	1.45 ± 0.1	1.13 ± 0.0	1.33 ± 0.1	No
2-butanone	600	600	2.79 ± 0.4	3.47 ± 0.4	3.54 ± 0.1	4.41 ± 0.7	No
1-penten-3-one	687	687	0.55 ± 0.1	0.74 ± 0.1	0.65 ± 0.0	0.50 ± 0.0	No
2-pentanone	688	695	2.76 ± 0.1	2.52 ± 0.3	2.69 ± 0.1	2.70 ± 0.3	No
2-pentenal (*E*)-	756	748	0.62 ± 0.0 *^b^*	1.10 ± 0.1 *^a^*	0.61 ± 0.0 *^b^*	0.51 ± 0.1 *^b^*	*
3-hexenal (*Z*)	797	795	10.3 ± 0.5 *^a^*	6.44 ± 0.5 *^b^*	2.78 ± 0.1 *^c^*	2.67 ± 0.3 *^c^*	**
hexanal	799	801	51.4 ± 2.2 *^a^*	49.2 ± 3.0 *^a^*	18.6 ± 0.1 *^b^*	18.3 ± 1.2 *^b^*	**
2,4-hexadienal (*E*,*E*)-	911	916	12.03 ± 0.8 *^a^*	11.3 ± 1.1 *^a^*	5.28 ± 0.3 *^b^*	5.24 ± 0.3 *^b^*	**
2-heptenal (*E*)-	960	964	1.47 ± 0.2 *^b^*	3.40 ± 0.1 *^a^*	3.16 ± 0.0 *^a^*	2.83 ± 0.1 *^a^*	**
benzaldehyde	972	961	5.03 ± 0.2 *^d^*	25.5 ± 0.4 *^a^*	13.7 ± 0.6 *^d^*	22.0 ± 0.3 *^c^*	**
6-methyl-5-hepten-2-one	988	988	4.03 ± 0.1 *^c^*	10.7 ± 0.3 *^b^*	13.3 ± 0.5 *^a^*^,*b*^	14.8 ± 0.8 *^a^*	**
phenylacetaldehyde	1055	1047	0.29 ± 0.1 *^b^*	1.01 ± 0.1 *^a^*	1.14 ± 0.0 *^a^*	1.05 ± 0.0 *^a^*	*
2-octenal (*E*)-	1063	1063	5.69 ± 0.2 c	9.54 ± 0.0 *^b^*	9.88 ± 0.2 *^a^*^,*b*^	10.7 ± 0.1 *^a^*	**
nonanal	1107	1102	3.63 ± 1.8	6.45 ± 0.6	4.69 ± 0.4	4.98 ± 0.4	No
***esters***							
methyl acetate *^t^*	<600	522	0.22 ± 0.0	0.21 ± 0.0	0.34 ± 0.0	0.28 ± 0.1	No
ethyl acetate	615	628	15.2 ± 0.4	15.9 ± 1.5 *^b^*	20.9 ± 0.4 *^a^*^,*b*^	23.3 ± 1.4 *^a^*	*
propyl acetate	715	695	0.50 ± 0.0 *^b^*	0.59 ± 0.1 *^b^*	0.64 ± 0.0 *^a^*^,*b*^	1.02 ± 0.1 *^a^*	*
methyl butanoate	724	710	1.00 ± 0.1	0.81 ± 0.1	0.88 ± 0.0	0.84 ± 0.1	No
butyl acetate	812	802	8.02 ± 0.1 *^b^*	9.52 ± 0.9 *^b^*	12.43 ± 0.4 *^a^*	17.63 ± 1.2 *^a^*	*
2-methyl butyl acetate	877	880	24.5 ± 0.6 *^b^*	36.7 ± 2.7 *^a^*^,*b*^	40.8 ± 1.2 *^a^*	49.3 ± 3.3 *^a^*	*
pentyl acetate	911	926	1.04 ± 0.1 *^d^*	3.16 ± 0.1 *^c^*	5.72 ± 0.0 *^b^*	7.22 ± 0.3 *^a^*	**
ethyl-3-hydroxy-butanoate	935	945	2.75 ± 0.1 *^b^*	2.93 ± 0.1 *^a^*	3.20 ± 0.3 *^a^*	1.58 ± 0.1 *^a^*	*
butyl butanoate	995	1002	2.03 ± 0.1 *^b^*	8.17 ± 0.8 *^a^*	7.15 ± 0.1 *^a^*	7.59 ± 0.7 *^a^*	*
3-hexen-1-yl acetate (*Z*)	1001	996	1.04 ± 0.4 *^d^*	11.5 ± 0.5 *^c^*	22.6 ± 0.4 *^b^*	29.8 ± 2.2 *^a^*	**
2-hexen-1-yl acetate (*E*)	1013	1017	5.83 ± 2.1 *^c^*	195 ± 9.6 *^b^*	244 ± 2.7 *^a^*	237 ± 12.4 *^a^*^,*b*^	**
hexyl acetate	1011	1011	18.5 ± 1.4 *^c^*	153 ± 9.3 *^b^*	279 ± 5.7 *^a^*	315 ± 20.7 *^a^*	**
2-methyl butyl butanoate	1042	1041	3.88 ± 0.6 *^b^*	11.0 ± 0.9 *^a^*	12.3 ± 0.3 *^a^*	10.8 ± 1.1 *^a^*	*
methyl octanoate	1123	1129	0.18 ± 0.1 *^b^*	2.10 ± 0.6 *^a^*	0.57 ± 0.1 *^a^*^,*b*^	0.41 ± 0.0 *^a^*^,*b*^	*
hexyl-2-methylpropanoate	1147	1138	0.30 ± 0.0 *^b^*	1.92 ± 0.1 *^a^*	2.23 ± 0.0 *^a^*	2.54 ± 0.3 *^a^*	**
hexyl-2-methyl butanoate	1238	1236	9.27 ± 0.8 *^c^*	65.4 ± 0.1 *^b^*	104 ± 0.4 *^a^*	101 ± 5.9 *^a^*	**
pentyl hexanoate	1287	1282	0.29 ± 0.0 *^b^*	3.01 ± 0.0 *^a^*	3.53 ± 0.1 *^a^*	3.20 ± 0.3 *^a^*	**
butyl octanoate	1387	1393	0.63 ± 0.1 *^c^*	3.06 ± 0.1 *^b^*	4.45 ± 0.1 *^a^*	4.21 ± 0.0 *^a^*	**
hexyl hexanoate	1386	1386	4.19 ± 0.3 *^c^*	28.7 ± 1.5 *^b^*	48.7 ± 1.7 *^a^*	48.5 ± 0.6 *^a^*	**
3-methylbutyl octanoate	1451	1450	0.16 ± 0.1 *^c^*	0.67 ± 0.0 *^b^*^,*c*^	1.43 ± 0.2 *^a^*	1.11 ± 0.1 *^a^*^,*b*^	*
methyljasmonate	1672	1647	1.29 ± 0.2	0.89 ± 0.1	1.38 ± 0.2	1.15 ± 0.0	No
***terpenoids***							
2-methyl 1,3-pentadiene (*E*)- *^t^*	669	n/a	0.12 ± 0.0 *^b^*	0.88 ± 0.1 *^a^*	0.87 ± 0.1 *^a^*	1.11 ± 0.1 *^a^*	*
linalool oxide isomer I *^t^*	1084	n/a	11.6 ± 0.6 *^b^*	22.2 ± 1.6 *^a^*	26.8 ± 0.0 *^a^*	26.9 ± 1.3 *^a^*	*
linalool oxide isomer II *^t^*	1099	n/a	11.5 ± 0.5 *^c^*	19.9 ± 0.5 *^b^*	23.4 ± 0.6 *^a^*	23.2 ± 0.6 *^a^*	**
β-damascenone	1408	1400	4.06 ± 0.2	4.25 ± 0.8	4.50 ± 0.9	5.72 ± 0.3	No
β-ionone	1511	1493	4.74 ± 0.4 *^b^*	7.25 ± 0.1 *^a^*	6.27 ± 0.1 *^a^*	7.32 ± 0.1 *^a^*	*
α-farnesene	1517	1508	21.4 ± 13.5	32.6 ± 4.5	60.0 ± 9.6	48.0 ± 4.4	No
***miscellaneous***							
2-ethylfuran	703	702	1.52 ± 0.2 *^a^*	1.28 ± 0.1 *^a^*^,*b*^	0.84 ± 0.1 *^b^*	0.73 ± 0.0 *^b^*	*
2-pentylfuran	995	991	0.67 ± 0.1	1.86 ± 0.5	1.59 ± 0.4	1.11 ± 0.3	No
hexanal dimethyl acetal	978	980	6.95 ± 0.9 *^a^*	7.02 ± 0.4 *^a^*	1.81 ± 0.3 *^b^*	1.90 ± 0.3 *^b^*	*

*^a^*^,*b*,*c*,*d*^ Different superscript letters in the same row indicate statistically significant differences among the samples obtained from ANOVA followed by post hoc pairwise comparison using Tukey’s HSD (*p* < 0.05). *^t^* tentative identification, identification based on the mass spectrum alone; n/a no RI on DB5 could be found in the literature. ^§^ linear temperature programmed retention indices determined in the experiments on a semi-polar column ^$^ linear temperature programmed retention indices obtained from databases on a semi-polar column from databases (in-house database built with authentic reference compounds; www.flavornet.org; www.odour.or.uk; Nist Webbook; last access 27 September 2021).

**Table 2:** Basic analytical parameters of the five different apple wines AW1 to AW5.

	Ethanol [%*v*/*v*]	Residual Sugar [g L^−1^]	Titratable Acid ^§^ [g L^−1^]	Malic Acid [g L^−1^]	Lactic Acid [g L^−1^]
AW1	7.4	<1.7	6.2	7.0	n.d.
AW2	7.3	2.5	6.8	7.4	n.d.
AW3	7.4	8.3	5.9	2.2	2.3
AW4	7.3	3.0	6.8	7.2	n.d.
AW5	7.2	8.1	5.8	2.1	2.3

^§^ expressed as tartaric acid equivalents g L^−1^; n.d. not detectable.

**Table 3:** Volatile compounds of the apple wines AW1 to AW5 that were produced with different fermentations strategies; quantities are given as mean relative concentrations [µg L^−1^] (*n* = 4) collected from the headspace using HS-SPME GC-MS calculated by comparison of the peak areas with that of the internal standard 2-octanol with a response factor of 1; SD standard deviation [g L^−1^]; * significant difference among all approaches (*p* ≤ 0,05%), ** highly significant difference among all approaches (*p* ≤ 0.001%), AW apple wine.

Compound	RI [DB5] _exp_^§^	RI [DB5] _lit._^$^	AW1 Mean ± SD [µg L^−1^]	AW2 Mean ± SD [µg L^−1^]	AW3 Mean ± SD [µg L^−1^]	AW4 mean ± SD [µg L^−1^]	AW5 mean ± SD [µg L^−1^]	Significance
***acids***								
3-methyl butanoic acid	823	835	8.0 ± 1.2 *^c^*	12.1 ± 0.8 *^a^*^,*b*^	13.6 ± 0.6 *^a^*	11.25 ± 0.2 *^b^*	13.2 ± 1.1 *^a^*^,*b*^	**
2-methyl butanoic acid	835	846	10.1 ± 2.1 *^c^*	18.6 ± 0.3 *^b^*	30.7 ± 2.5 *^a^*	17.30 ± 1.7 *^b^*	27.4 ± 2.5 a	**
hexanoic acid	969	980	403 ± 45.7 *^a^*	357 ± 22 *^a^*	105 ± 8.7 *^b^*	375.47 ± 12.4 *^a^*	102 ± 12 *^b^*	**
octanoic acid	1164	1178	721 ± 158 *^a^*	889 ± 177 *^a^*	169 ± 10.3 *^b^*	977.1 ± 211.4 *^a^*	152 ± 15 *^b^*	**
***alcohols***								
2-methyl-1-butanol	719	730	437 ± 38.3 *^c^*	580 ± 16 *^b^*	800 ± 84 *^a^*	569 ± 22 *^b^*	777 ± 15 *^a^*	**
3-methyl-1-butanol	722	743	3526 ± 230	3 812 ± 417	3 648 ± 315	3 936 ± 87	3 864 ± 343	no
1-pentanol	753	766	4.0 ± 0.2 *^c^*	6.9 ± 0.3 *^b^*	11.0 ± 1.2 *^c^*	6.27 ± 0.6 *^b^*	10.5 ± 0.3 *^c^*	**
2,3-butanediol	779	773	54.0 ± 18.7	51.1 ± 27.9	24.7 ± 4.5	42.77 ± 13.5	18.3 ± 3.8	no
3-ethoxy-1-propanol	836	816	58.7 ± 2.8 *^a^*	27.7 ± 1.7 *^b^*	5.9 ± 3.2 *^d^*	18.73 ± 1.5 *^c^*	4.1 ± 1.6 *^d^*	**
3-hexen-1-ol (*E*)	848	858	3.8 ± 0.7 *^c^*	17.2 ± 0.8 *^b^*	22.0 ± 0.7 *^a^*	16.14 ± 0.6 *^b^*	21.3 ± 0.8 *^a^*	**
1-hexanol	865	867	434 ± 31.4 *^c^*	1 158 ± 48 *^b^*	1 388 ± 78 *^a^*	1 097 ± 23.8 *^b^*	1 388 ± 59 *^a^*	**
1-heptanol	968	970	0.9 ± 0.2 *^b^*	1.5 ± 0.2 *^b^*	19.6 ± 0.3 *^a^*	1.38 ± 0.1 *^b^*	18.9 ± 0.6 *^a^*	**
2-methyl-6-hepten-1-ol	992	994	49.6 ± 2.0 *^c^*	54.6 ± 2.3 *^c^*	79.4 ± 5.0 *^a^*	50.8 ± 2.3 *^c^*	71.6 ± 1.8 *^b^*	**
3-ethyl-4-methylpentan-1-ol	1024	1020	0.3 ± 0.1 *^c^*	3.5 ± 0.1 *^b^*	4.2 ± 0.3 *^c^*	3.47 ± 0.1 *^b^*	4.1 ± 0.1 *^c^*	**
2-ethylhexanol	1029	1029	4.8 ± 0.4	5.8 ± 1.0	5.6 ± 0.2	5.98 ± 0.5	5.8 ± 0.2	no
benzyl alcohol	1041	1032	1.2 ± 0.2 *^c^*	5.1 ± 0.6 *^c^*	76.4 ± 3.4 *^a^*	4.84 ± 0.4 *^c^*	67.7 ± 2.8 *^b^*	**
1-octanol	1069	1069	4.3 ± 0.5 *^d^*	18.1 ± 0.7 *^c^*	33.9 ± 0.8 *^a^*	17.19 ± 0.8 *^c^*	30.9 ± 1.3 *^b^*	**
phenylethyl alcohol	1127	1113	629 ± 48 *^a^*	727 ± 55 *^a^*^,*b*^	618 ± 17 *^b^*	611 ± 39 *^b^*	558 ± 20 *^b^*	*
***carbonyls***								
benzaldehyde	971	961	6.8 ± 0.9 ^c^	11.1 ±1.3 *^b^*	19.5 ±1.6 *^a^*	10.3 ±0.3 *^b^*	18.5 ± 0.8 *^a^*	**
nonanal	1106	1101	8.5 ± 3.4	7.3 ± 3.2	6.6 ± 1.3	7.0 ± 5.8	5.8 ± 1.2	no
2-butanone	601	600	9.5 ± 1.3	8.3 ±1.3	7.5 ±1.1	9.8 ±0.9	9.1 ±1.2	No
***esters***								
ethyl acetate	614	628	315 ± 63 *^a^*	311 ± 52 *^a^*	143 ± 32 *^b^*	309 ± 20 *^a^*	168 ± 20 *^b^*	**
ethyl propanoate	692	706	1.4 ± 0.4 *^b^*	2.5 ± 0.3 *^a^*^,*b*^	3.3 ± 0.8 *^a^*	2.0 ± 0.2 *^b^*	3.12 ± 0.3 *^a^*	**
propyl acetate	694	695	4.5 ± 1.0 *^a^*	3.9 ± 0.4 *^a^*	1.2 ± 0.2 *^b^*	3.7 ± 0.4 *^a^*	1.2 ± 0.2 *^b^*	**
ethyl butanoate	791	808	31.3 ± 8.6 *^a^*	36.5 ± 3.1 *^a^*	6.4 ± 1.6 *^b^*	30.5 ± 2.2 *^a^*	5.5 ± 2.2 *^b^*	**
butyl acetate	805	802	29.4 ± 5.4 *^a^*	26.0 ± 1.7 *^a^*	9.9 ± 0.5 *^b^*	23.2 ± 1.0 *^a^*	11.4 ± 0.4 *^b^*	**
ethyl 2-methyl butanoate	846	850	0.9 ± 0.3 *^b^*	2.1 ± 0.2 *^b^*	5.9 ± 1.3 *^a^*	1.5 ± 0.2 *^b^*	5.6 ± 0.9 *^a^*	**
2-methylbutyl acetate	876	880	114 ± 33 *^a^*	95.4 ± 6.3 *^a^*	13.1 ± 1.2 *^b^*	81.7 ± 7.2 *^a^*	15.7 ± 1.9 *^b^*	**
3-methylbutyl acetate	873	876	1028 ± 254 *^a^*	770 ± 46 *^a^*^,*b*^	106 ± 9.5 *^c^*	670 ± 48 *^b^*	121 ± 12 *^c^*	**
methyl hexanoate	923	936	7.5 ± 2.6 *^c^*	18.7 ± 2.1 *^a^*^,*b*^	12.8 ± 1.9 *^b^*^,*c*^	19.7 ± 3.3 *^a^*	13.4 ± 1.6 *^b^*^,*c*^	**
ethyl-3-hydroxy butanoate	934	945	1.0 ± 0.1 *^c^*	2.1 ± 0.2 *^b^*	2.4 ± 0.1 *^a^*	1.0 ± 0.1 *^c^*	1.2 ± 0.2 *^c^*	**
ethyl hexanoate	997	999	956 ± 258 *^a^*	980 ± 47 *^a^*	587 ± 15 *^b^*	846 ± 46 *^a^*	275 ± 37 *^b^*	**
hexyl acetate	1010	1011	1072 ± 290 *^a^*	795 ± 44 *^a^*^,*b*^	47.0 ± 1.5 *^c^*	657 ± 32 *^b^*	55.4 ± 6.3 *^c^*	**
isoamyl lactate	1071	1065	0.3 ± 0.1 *^c^*	0.2 ± 0.1 *^c^*	23.8 ± 1.4 *^a^*	0.3 ± 0.1 *^c^*	20.5 ± 1.1 *^b^*	**
methyl octanoate	1123	1129	216 ± 78 *^a^*	233 ± 63 *^a^*	69.9 ± 17.2 *^b^*	241 ± 69 *^a^*	66.8 ± 15.6 *^b^*	*
ethyl benzoate	1183	1170	0.7 ± 0.1 *^c^*	1.4 ± 0.0 *^c^*	12.8 ± 0.3 *^a^*	1.6 ± 0.1 *^c^*	10.8 ± 0.7 *^b^*	**
ethyl octanoate	1196	1194	2083 ± 479 *^a^*	2148 ± 123 *^a^*	561 ± 46 *^b^*	1 994 ± 101 *^a^*	483 ± 77 *^b^*	**
hexyl-2-methylbutanoate	1238	1234	0.5 ± 0.1 *^b^*	3.1 ± 0.1 *^b^*	36.9 ± 2.8 *^a^*	2.2 ± 0.2 *^b^*	32.4 ± 4.9 *^a^*	**
ethylphenyl acetate *^t^*	1255	n/a	0.8 ± 0.1 *^c^*	2.0 ± 0.2 *^b^*	11.6 ± 0.5 *^a^*	1.9 ± 0.2 *^b^*	11.4 ± 0.6 *^a^*	**
phenethyl acetate	1269	1256	222 ± 16.8 *^a^*	140.1 ± 6.0 *^b^*	16.7 ± 0.9 *^d^*	113 ± 4.2 *^c^*	15.5 ± 0.4 *^d^*	**
methyl decanoate	1324	1324	387 ± 81 *^a^*	368 ± 81 *^a^*	56.8 ± 13 *^b^*	363 ± 70 *^a^*	51.3 ± 11 *^b^*	**
2-methylpropyl octanoate	1348	1345	6.0 ± 1.7 *^a^*	6.3 ± 0.8 *^a^*	1.1 ± 0.2 *^b^*	6.4 ± 1.1 *^a^*	1.0 ± 0.2 *^b^*	**
ethyl decanoate	1395	1392	2177 ± 445 *^a^*	1832 ± 160 *^a^*	279 ± 41 *^b^*	1 941 ± 245 *^a^*	230 ± 40 *^b^*	**
3-methylbutyl octanoate	1448	1450	104 ± 30 *^a^*	88.7 ± 10.8 *^a^*	9.2 ± 1.6 *^b^*	94.8 ± 18.1 *^a^*	7.2 ± 1.2 *^b^*	**
methyl dodecanoate	1525	1526	276 ± 16.6 *^a^*	291 ± 41 *^a^*	61.8 ± 7.0 *^b^*	285 ± 26.2 *^a^*	52.8 ± 5.2 *^b^*	**
hexyl octanoate	1584	1571	3.3 ± 1.2 *^b^*	8.5 ± 1.0 *^a^*	0.9 ± 0.2 *^b^*	9.0 ± 2.2 *^a^*	0.8 ± 0.2 *^b^*	**
ethyl dodecanoate	1594	1597	547 ± 194 *^a^*	395 ± 61 *^a^*	53.3 ± 12.2 *^b^*	533 ± 134 *^a^*	43.4 ± 10.4 *^b^*	**
3-methylbutyl decanoate	1647	1649	108.9 ± 31.2 *^a^*	96.0 ± 10.5 *^a^*	6.4 ± 1.5 *^b^*	109 ± 18.4 *^a^*	5.1 ± 1.1 *^b^*	**
2-methylbutyl decanoate	1651	1647	16.4 ± 5.4 *^a^*	17.0 ± 2.1 *^a^*	2.5 ± 0.2 *^b^*	21.5 ± 4.5 *^a^*	2.3 ± 0.2 *^b^*	**
2-phenylethyl hexanoate	1664	1650	5.4 ± 0.7 *^a^*	4.5 ± 0.2 *^b^*	0.6 ± 0.1 *^c^*	3.8 ± 0.2 *^b^*	0.6 ± 0.1 *^c^*	**
ethyl tetradecanoate	1794	1794	26.9 ± 8.8 *^a^*	17.7 ± 2.6 *^a^*^,*b*^	3.6 ± 1.6 *^b^*	26.1 ± 5.8 *^a^*	5.7 ± 0.8 *^b^*	**
3-methylbutyl dodecanoate	1848	1844	6.8 ± 1.6 *^ab^*	4.8 ± 0.9 *^b^*	0.9 ± 0.2 *^c^*	7.2 ± 0.7 *^a^*	0.8 ± 0.1 *^c^*	**
methyl hexadecanoate	1927	1933	132 ± 32	135 ± 38	86 ± 12	147 ± 41	89 ± 19	no
ethyl-9-hexadecenoate	1979	1977	7.2 ± 1.2 *^c^*	16.6 ± 1.8 *^a^*	4.2 ± 0.7 *^d^*	13.3 ± 0.9 *^b^*	2.9 ± 0.2 *^d^*	**
ethyl hexadecanoate	1994	1978	53.1 ± 3.1 *^a^*	35.7 ± 4.3 *^b^*	25.8 ± 2.2 *^c^*	53.0 ± 5.3 *^a^*	24.2 ± 1.3 *^c^*	**
***terpenoids***								
linalool oxide (furanoid)	1083	1073	5.9 ± 0.1 *^c^*	9.0 ± 0.5 *^a^*	9.2 ± 0.5 *^a^*	7.4 ± 0.0 *^b^*	8.1 ± 0.2 *^b^*	**
linalool	1103	1101	3.6 ± 0.5 *^d^*	5.5 ± 0.1 *^a^*	4.8 ± 0.1 *^b^*^,*c*^	5.1 ± 0.1 *^b^*^,*c*^	4.4 ± 0.2 *^a^*^,*b*^	**
α-farnesene	1517	1508	2.0 ± 0.3 *^c^*	7.3 ± 0.5 *^a^*^,*b*^	9.2 ± 1.7 *^a^*	6.3 ± 0.8 *^b^*	8.1 ± 1.0 *^a^*^,*b*^	**

*^a^*^,*b*,*c*,*d*^ Different superscript letters in the same row indicate statistically significant differences among the samples obtained from ANOVA followed by post hoc pairwise comparison using Tukey’s HSD (*p* < 0.05). *^t^*Tentative identification, identification based on the mass spectrum alone; n/a no RI on DB5 could be found in the literature. ^§^ Linear temperature programmed retention indices determined in the experiments on a semi-polar column. ^$^ Linear temperature programmed retention indices obtained from databases on a semi-polar column from databases (in-house database built with authentic reference compounds; www.flavornet.org; www.odour.or.uk; Nist Webbook; 27 September 2021).

## Data Availability

Data are contained within the article.
